# Selection for Function in Complex Distributed Pathological Systems

**DOI:** 10.1111/eva.70202

**Published:** 2026-02-02

**Authors:** Frédéric Thomas, Antoine M. Dujon, Daniel Vaiman, Gerard Eberl, Catherine Alix‐Panabières, Pascal Pujol, Beata Ujvari, Jordan Meliani, Aurora M. Nedelcu, Jean‐Pascal Capp

**Affiliations:** ^1^ CREEC/CANECEV, MIVEGEC (CREES) Department University of Montpellier, CNRS, IRD Montpellier France; ^2^ School of Life and Environmental Sciences, Deakin University Waurn Ponds Victoria Australia; ^3^ Institut Cochin, U1016, INSERM, UMR8104 CNRS, Université de Paris Paris France; ^4^ Microenvironment & Immunity Unit, Institut Pasteur Paris France; ^5^ Laboratory of Rare Human Circulating Cells and Liquid Biopsy (LCCRH), University Medical Centre of Montpellier Montpellier France; ^6^ European Liquid Biopsy Society (ELBS) Hamburg Germany; ^7^ Oncogenetic Department University Medical Centre of Montpellier Montpellier France; ^8^ Department of Biology University of New Brunswick Fredericton New Brunswick Canada; ^9^ Toulouse Biotechnology Institute, University of Toulouse, INSA, CNRS, INRAE Toulouse France

## Abstract

Pathological processes are often conceptualized as localized phenomena anchored in a primary tumor, a focal lesion, or a single organ. However, growing evidence indicates that many diseases persist and progress as complex distributed systems, maintained by interactions among multiple sites. Building on the emerging framework of selection for function, which can be applied to understand the evolutionary persistence of both replicating and non‐replicating entities, we propose that metastases, amyloidoses, fibroses, autoimmune syndromes, granulomatous diseases, and multifocal reproductive disorders can all be understood as complex evolving pathological systems within individuals. In these contexts, local units such as metastatic nodules, amyloid plaques, or fibrotic foci act as semi‐autonomous entities, yet achieve collective persistence through systemic flows, feedback loops, and network‐level interactions, where local structuration gives rise to systemic effects. At certain points, lesions that produce mediators can trigger systemic alterations that, in turn, favor the emergence and persistence of additional lesions. This creates a vicious cycle in which local and systemic dynamics reinforce one another, helping these specific pathological networks to overcome host defense mechanisms and persist (i.e., be ‘selected’ via differential persistence). This perspective unifies seemingly disparate conditions under the principle of system persistence, reframing pathology as an emergent organizational property of a pathological system rather than as isolated local breakdowns of organismal components. It also carries important implications for evolutionary medicine, suggesting a taxonomy of diseases that distinguishes localized from distributed functional pathologies. Clinically, it underscores the need to go beyond focal interventions, advocating instead for therapies that disrupt pathological connectivity, destabilize network coherence, and monitor systemic biomarkers of disease persistence. Recognizing the role of selection for function in the emergence and persistence of complex pathological systems opens new avenues for both theoretical integration and therapeutic innovation in evolutionary medicine.

## 1 Introduction

Despite ongoing debate (e.g., Lynch 2025; Jaeger 2024), the concept of *selection for function*, introduced by (Wong et al. 2023), is gaining ground as a unifying evolutionary framework that can be applied to both replicating and non‐replicating systems. It sheds light on a wide range of evolutionary dynamics, especially in non‐replicative living entities (see Box 1 and Thomas, Hamant, et al. 2025). In the context of health, it was initially proposed by Thomas et al. (2024) to account for tumor progression and persistence. Indeed, this framework highlights how groups of cells can acquire and sustain collective functional properties that ensure the survival and expansion of the pathological entity (i.e., the tumor), even when such functions are detrimental to the host organism (Thomas et al., submitted). This perspective on tumors carries important therapeutic implications, underscoring the need to view cancer as an adaptive complex system rather than a mere collection of mutated cells with different individual fitnesses (Capp et al. 2021, 2023). By recognizing and interfering with the functional cohesion of tumor cell groups, this framework paves the way for innovative treatment paradigms that aim to destabilize the pathological entity as a whole (Thomas, Capp, et al. 2025). More recently, the same reasoning has been extended to senescence‐related conditions (Dujon et al. 2025), such as atherosclerotic plaques or lipomas, where age‐related relaxation of selective pressures at the organismal level enables some pathological functional assemblies within individuals to organize, persist and thrive (Box 2). Strikingly, *selection for function* may also help explain disorders arising prior to the post‐reproductive phase, such as endometriosis, where pathological structures sustain themselves through autonomous networks and resource flows (Asselin et al. submitted, Box 3).

BOX 1 **The concept of selection for function.**

*Selection for function* is an emerging evolutionary framework proposed by Wong et al. 2023, which explains how certain structural or organizational systems, biological or not, can persist and evolve over time, regardless of their reproductive capacity. This concept applies not only to biological systems but also to abiotic systems (e.g., mineral formations Hazen and Wong 2024), technological systems, and ecological networks. The framework is an extension of classical Darwinian selection, which operates through heritable differential reproductive success of self‐replicating entities, by allowing selection to also act on non‐reproducing systems and favor configurations that enhance the persistence, robustness, and adaptability of a system as a whole.

A system can evolve through selection for function if it meets the following criteria: it consists of many interacting components capable of forming a wide range of configurations; mechanisms exist that generate diverse configurations; and certain configurations are preferentially selected due to their contribution to one or more of these ‘functions’:

- **Static persistence**: whereby some configurations remain stable because they resist degradation or disruption.
- **Dynamic persistence**: through which systems adapt to perturbations, increasing their resilience without replication.
- **Novelty generation**: which allows new configurations to emerge that confer greater system‐level stability or functionality.

In distributed pathological systems, the three conceptual equivalencies of selection for function take concrete forms. The interacting components correspond to autonomous pathological units (e.g., metastatic nodules, amyloid deposits, fibrotic foci). The generation of multiple configurations arises from their connectivity through systemic flows, such as circulating cells, misfolded proteins, immune mediators, hormones, or mechanical signals, which allows new spatial and functional arrangements to emerge across tissues. Selection for function operates through differential persistence of distributed organizations: configurations that enhance system‐level stability, propagation, or resistance to host defenses and therapies are preferentially maintained, even when they are detrimental to the organism.

Within organisms, non‐replicative structures such as atherosclerotic plaques, amyloid aggregates, or benign tumors may evolve toward increasingly organized forms via selection for function, particularly when higher‐level constraints (e.g., organismal fitness control) decline with age (see Box 2). Sometimes, specific contexts can promote these pathological processes earlier during life (see Box 3). By shifting the evolutionary focus from replication and heritability to persistence, selection for function offers a unifying principle that expands the scope of evolutionary theory beyond gene‐centric models. It provides a novel lens through which to understand the evolution of certain diseases.

BOX 2 **Selection for function and chronic diseases of aging.**

Classical evolutionary theories of aging, such as mutation accumulation, antagonistic pleiotropy, or the disposable soma theory, explain aging, and consequently, late‐life diseases as consequences of the declining force of natural selection with age. These models describe why deleterious alleles and physiological trade‐offs with late‐in life negative effects accumulate in populations (i.e., the “selection shadow”), but they often treat age‐related pathologies as largely passive outcomes of weakened evolutionary constraints. However, this view can be overly simplified. In many species, selection acts most strongly at specific stages of the life cycle, such as between birth and weaning, and late‐life vulnerabilities can also reflect the cumulative effects of developmental “memory,” including epigenetic modifications, long‐term changes in tissue organization, neural circuitry, or even past environmental or physiological stresses. These trajectories can shape the emergence of chronic diseases later in life, independent of age‐related declines in selection.

A complementary view has been recently proposed by Dujon et al. (2025): many chronic diseases of aging may reflect the action of within‐organism selection for function at lower organizational levels. Selection for function provides a new lens to understand why non‐replicative pathological entities, such as tumors, atherosclerotic plaques, or amyloid aggregates, often become more organized, structured, and resistant over time. However, these increasingly coherent configurations do not necessarily represent adaptive responses. Rather, they may emerge as consequences of prior life trajectories, long‐term microenvironmental conditions, or earlier physiological and developmental “settings” that shape how tissues respond to damage, inflammation, or stress. In such contexts, selection for function highlights how pre‐existing constraints and histories can channel the self‐organization of pathological structures toward persistent, self‐stabilizing states. Early in life, strong organism‐level selection constrains these processes, actively clearing unstable or deleterious structures. With aging, however, clearance mechanisms weaken and selective constraints at the organismal level decline, allowing certain configurations to persist and consolidate through selection for function at the lower level. For example, fibrous caps in atherosclerotic plaques, compact amyloid and tau aggregates in Alzheimer's disease, or stable tumor architectures represent organizational states that have been “selected for” because they resist degradation and adapt to local conditions, even though they compromise organismal health.

This framework shifts the interpretation of chronic diseases from passive degeneration to active internal evolutionary dynamics, where selection‐like processes operate within tissues. Importantly, selection for function is distinct from both individual‐level and group selection: it does not require replication or heredity, but instead filters configurations that persist under given constraints.

Therapeutically, this insight opens new perspectives. Rather than focusing exclusively on eliminating cells, molecules, or symptoms, interventions could aim to destabilize the functional organization of persistent disease structures, preventing them from reaching resilient, self‐sustaining configurations. In cancer, this may mean targeting the collective properties of tumors (their Group Phenotypic Composition; Capp et al. 2021); in atherosclerosis, disrupting fibrous cap stabilization; and in Alzheimer's disease, preventing amyloid or tau from consolidating into resilient aggregates. Importantly, the mechanisms that stabilize these pathological structures often co‐opt the very same forces that normally maintain tissue integrity—such as damage repair pathways, structural reinforcement responses, or homeostatic signaling. Under conditions of aging or chronic stress, these mechanisms can become dysregulated, expressed in atypical contexts or locked into maladaptive configurations. Thus, the strategies proposed here aim to intervene in processes that, while essential for healthy tissue maintenance, can inadvertently sustain persistent pathological structures in aging tissues. Such an approach may complement existing geroscience strategies by focusing not only on clearing damage but also on destabilizing the self‐reinforcing architectures that enable chronic diseases to persist.

By shifting the evolutionary focus from reproduction to persistence, selection for function broadens evolutionary theory and provides a unifying framework for understanding why chronic diseases become more structured, complex, and resistant with age.

BOX 3 While persistent, organized pathologies such as tumors, fibrotic nodules, or amyloid plaques are often associated with aging, growing evidence indicates that similar processes can also shape early‐life diseases. This challenges the classical view that robust pathological structures emerge only after reproduction, when organism‐level selection weakens.

To explain this paradox, it has been proposed (Asselin et al. submitted) that intra‐organismal selection for function can sometimes operate *before aging*. Rather than being mere developmental anomalies, some early‐onset pathologies may reflect eco‐evolutionary dynamics within tissues.

Three non‐mutually exclusive mechanisms can explain how selection for function acts in early life:

1. **Early‐life adaptive benefits**: Local tissue organization may initially serve beneficial purposes, such as wound healing, infection containment, or structural reinforcement. However, these organized responses (e.g., fibrotic nodules, granulomas) may persist maladaptively once established.
2. **Limited or context‐dependent costs**: Some stable pathological structures impose little or no reproductive penalty early in life. Examples include benign tumors, calcifications, or endometriotic lesions, which can persist for years without severely impacting host fitness.
3. **Novel environmental pressures**: Modern exposures (pollutants, endocrine disruptors, dietary changes, chronic inflammation) reshape tissue microenvironments and create conditions that favor stable pathological organizations earlier than expected in evolutionary history.

Examples include granulomas that persist for decades in tuberculosis or leprosy, endometriosis with ectopic but stable endometrial tissue growth, biofilms resistant to immune clearance, and benign tumors or calcifications that reinforce tissue architecture. In each case, microenvironmental dynamics, such as altered extracellular matrix, immune evasion, or metabolic gradients, favor persistent configurations through selection for function. Notably, many of these processes can have beneficial or adaptive origins. Granulomas, for instance, initially serve to contain pathogens; biofilm‐like assemblies can enhance microbial survival in ways that may protect host‐associated communities; and fibrotic nodules or calcifications may arise as structural reinforcements following tissue damage. Selection for function thus helps explain how organizational features that are advantageous or protective in the short term can, under certain conditions, become locked into persistent configurations that ultimately contribute to chronic pathology.

This framework reframes early‐onset pathologies as products of local eco‐evolutionary dynamics rather than passive developmental errors. Importantly, it also suggests therapeutic strategies that directly target the organizational features enabling persistent pathological structures. These include destabilizing extracellular matrix and structural scaffolds, disrupting local communication networks (e.g., quorum sensing in biofilms), modulating microenvironmental conditions (pH, oxygen, nutrient gradients), and reducing chronic inflammation or environmental triggers. While some of these approaches already exist in clinical oncology or tissue‐targeted therapies—such as modifying blood flow, targeting stromal cells, or altering extracellular matrix properties—our framework unifies them under a common evolutionary rationale. By explicitly viewing tissues as eco‐evolutionary systems, it highlights why interventions that modify structural or microenvironmental features can be particularly effective at preventing pathological configurations from becoming self‐sustaining.

By targeting the functional organization rather than only individual cells, treatments could undermine the resilience of pathological structures and prevent their long‐term persistence. Thus, Selection for function does not only shape late‐life diseases; it can also act early, whenever tissue‐level dynamics favor organized, persistent configurations. Recognizing these dynamics broadens our understanding of disease origins and opens novel therapeutic avenues to prevent or control early‐life pathologies.

Despite these advances, most analyses still conceptualize pathological processes as localized phenomena, typically anchored in a primary tumor, a single organ, or a focal site of dysfunction. This bias has shaped both research and clinical strategies, which often aim to identify and target the focal point(s) of disease. Yet, growing evidence (see below) suggests that many pathologies are better understood as distributed systems, where multiple sites, lesions, or deposits interact, reinforce one another, and persist through emergent collective dynamics. In such cases, the disease is not the outcome of a single lesion or affected organ; rather, the pathology is maintained by network‐level interactions among different sites that favor system persistence through selection for function.

In this paper, we argue that extending the notion of selection for function to such complex pathological systems opens new conceptual grounds. In these cases, pathological coherence emerges from self‐organizing processes, where local units (e.g., tumors, deposits, granulomas, fibrotic nodules) interact through feedback loops, shared resources, or systemic signaling. These distributed pathologies resemble ecological networks or superorganisms, in which the persistence of the whole depends on connectivity and cooperation among its organizing parts, rather than on a central hub. Recognizing that selection for function can act on these decentralized systems has at least three major implications. First, it broadens the scope of evolutionary medicine by revealing common principles behind seemingly disparate conditions, from metastatic cancers to amyloidoses, autoimmune syndromes, and systemic fibroses. Second, it reframes pathology not only as a local breakdown in an organismal component, but also as a network phenomenon, where the key to its persistence lies in emergent functional integration across sites. Finally, it suggests novel therapeutic strategies: if pathological systems thrive through distributed connectivity, then breaking their communication, resource flows, or systemic coherence may prove as crucial as eliminating individual lesions. However, such approaches should be viewed as complementary to conventional local interventions, since individual lesions can also persist and evolve autonomously. Open questions remain as to whether lesions that stop contributing systemic mediators will disappear, persist as ‘cheaters,’ or continue their own dynamics independently.

In this paper, we propose to develop this perspective by examining how selection for function operates in distributed pathological systems favoring those that meet the requirements for such systems to persist. Using metastatic cancer as a central example, and drawing parallels with other multifocal and systemic diseases, we highlight the value of this framework for understanding the persistence of distributed pathologies and for inspiring new therapeutic approaches.

## 2 Metastatic Cancer as a Paradigm of Distributed Pathology

Metastasis has long been regarded as the ultimate expression of cancer progression, traditionally conceptualized as a cascade driven by the primary tumor (Gui and Bivona 2022). In this classical view, the primary tumor functions as the central hub: it sheds cells, which colonize distant organs, and these secondary sites remain subordinated to the dynamics of the original lesion. However, mounting evidence challenges this centralized narrative. Clinical and experimental studies now show that metastases can themselves release circulating tumor cells, seed additional metastatic sites, and even “re‐seed” the primary tumor, a process known as tumor self‐seeding (Aguirre‐Ghiso 2010; Liu et al. 2018; Kim et al. 2009). Such dynamics reveal that the metastatic system is not merely a hierarchy emanating from a single center, but rather a network of interconnected lesions. Within this network, each metastatic site operates as an autonomous functional (but not reproductive) unit, capable of growth, vascularization, and local adaptation. Yet, their persistence and expansion are reinforced through reciprocal interactions: cells, growth factors, and exosomes circulate between sites, maintaining systemic coherence (e.g., Wortzel et al. 2019; Guo et al. 2019). Importantly, this occurs in the absence of any program or central coordination. Although tumor‐derived exosomes and other systemic mediators can prepare metastatic niches and promote the growth of distant lesions, these processes arise from locally produced signals generated by individual lesions acting in parallel, rather than from any form of centralized control or inherited program. Each metastatic lesion produces its own molecular cues, and the global pattern of systemic conditioning emerges from the distributed contributions of multiple sites instead of a single organizing source. This raises the question of whether such interactions represent true cooperative interactions or rather a diffuse cross‐talk that functionally benefits the pathological network. Regardless, the functional integrity of the metastatic system emerges from distributed interactions among multiple lesions, resembling the organization of ecological networks or microbial biofilms. From the perspective of selection for function, metastases exemplify pathological entities that can interact and organize into many configurations that have the potential to form a higher‐level structure: a self‐organizing population of tumors (Thomas and Dujon 2025). Each lesion, including the primary tumor, benefits from being part of this network, as systemic signals and cellular exchanges enhance growth, survival, and therapeutic resistance. In the selection for function framework, the “fitness” (i.e., static and dynamic persistence as well as novelty generation) of the metastatic system arises not from the success of any single lesion, but from the collective performance of the network as a whole.

This distributed interpretation of metastasis carries profound clinical implications. In conventional oncology, the primary tumor is often prioritized for removal or control—not necessarily because it is viewed as the central organizer of the disease, but because it is assumed to play a key role in driving further dissemination or sustaining systemic progression, even once metastases are already present. However, if metastases operate as a decentralized pathological network, interventions focused predominantly on the primary lesion may fail to disrupt the systemic processes that maintain and propagate the metastatic ensemble. Instead, therapeutic strategies may need to target the connectivity and communication channels linking metastatic sites, by blocking circulating tumor cells, interfering with systemic signaling, or destabilizing the interdependence among lesions.

## 3 Other Distributed Pathological Systems

While metastatic cancer offers a striking paradigm of how pathological coherence can emerge from distributed interactions, it is not unique. Several other diseases display comparable dynamics, in which multiple lesions or deposits function as semi‐autonomous units that collectively can form a network that can be stabilized by selection acting at the system level. In each case, the pathology persists not because of a central hub, but because local structures interact and organize to sustain each other and the system through systemic flows of signals, resources, or feedback loops (Table 1).

**TABLE 1:** Distributed pathologies overview.

Pathology	Local autonomous unit	Mode of connectivity	Clinical consequence
Metastatic cancer	Metastatic nodule (proliferation, angiogenesis, local adaptation)	Circulating tumor cells, exosomes, growth factors	Network‐level tumor persistence, therapy resistance
Endometriosis/multifocal reproductive disorders	Ectopic endometrial lesion (vascularized, hormonally responsive)	Hormonal cycles, cytokines, vascular/inflammatory mediators	Recurrence, chronic pain, infertility, systemic inflammation
Amyloidoses/protein aggregation diseases	Amyloid plaque/fibril aggregate (structurally stable, prion‐like seeding)	Seeding by misfolded proteins, systemic or trans‐synaptic spread	Progressive neurodegeneration, multi‐organ involvement
Fibroses/systemic sclerosis	Fibrotic nodule with fibroblast–ECM feedback	Cytokines (TGF‐β, IL‐6), mechanical stress signals	Organ failure (lung, liver, kidney, skin), systemic sclerosis
Chronic autoimmune diseases (e.g., RA)	Inflamed joint niche (synovium with immune cell loops)	Autoantibodies, circulating T cells, systemic cytokines	Persistent systemic inflammation, joint destruction
Granulomatous diseases (TB, sarcoidosis)	Granuloma (macrophages, lymphocytes, giant cells around pathogen/irritant)	Immune signaling, systemic cytokines, pathogen dissemination	Chronic infection/inflammation, relapse, tissue destruction

### 3.1 Endometriosis and Multifocal Reproductive Disorders

Endometriosis illustrates how ectopic tissues can establish multiple foci of growth, often scattered across the peritoneum, ovaries, and other organs. Each lesion recruits vascularization, interacts with local immune cells, and sustains itself as a functional node (Burney and Giudice 2012; Zondervan et al. 2020). Yet these nodes are not independent: hormonal cycles, inflammatory mediators, and vascular networks create systemic links between lesions, ensuring the persistence of the disease as a distributed system (Bulun 2009; Vercellini et al. 2014). Several hypotheses have been proposed to explain these observations, such as the transport of mesenchymal stem cells (instrumental in fibrotic diseases) and their associated secreted factors by the peritoneal fluid (Zhang et al. 2019). Exchanges may be carried out through extracellular vesicles that have been shown to contain specific non‐coding RNA signatures (Khalaj et al. 2019). Importantly, cross‐talk among lesions, mediated by circulating cytokines, endocrine factors or regulatory RNAs, may reinforce the stability of the whole system, contributing to recurrence and resistance to therapy (Ahn et al. 2015; Saunders and Horne 2021). In this sense, when each lesion contributes to the global disruption that, in turn, promotes the persistence of all lesions, the process can be viewed as a form of emergent cooperation, albeit emerging without intentional coordination. Similar principles may apply to other multifocal reproductive pathologies, such as ovarian cysts or adenomyosis, where dispersed lesions form a functional ensemble without central control, collectively reshaping the tissue environment and sustaining chronic inflammation (Khan et al. 2010; Chapron et al. 2019). In some cases, their behavior even resembles that of ectopic tissue grafts: once established, lesions can maintain themselves autonomously while affecting distant sites through shared hormonal, inflammatory, or stromal signals.

### 3.2 Amyloidoses and Protein Aggregation Diseases

In amyloidoses, Alzheimer's disease, Parkinson's disease, Huntington's disease, and other proteinopathies, misfolded proteins aggregate into fibrillar structures in multiple brain regions or peripheral organs (Chiti and Dobson 2017; Knowles et al. 2014). Each deposit is structurally stable and self‐reinforcing, sustained by intermolecular β‐sheet interactions that confer remarkable resistance to clearance mechanisms (Goedert 2015; Caughey and Lansbury 2003). Crucially, pathology is not confined to isolated sites: soluble misfolded precursors and oligomeric seeds circulate systemically or spread trans‐synaptically, initiating new deposits in distant locations (Jucker and Walker 2013; Soto 2012). This prion‐like mechanism underlies the progressive dissemination of lesions across tissues and brain networks, representing a process of pathological spreading rather than cooperation in the strict sense. Nevertheless, once multiple deposits are established, their systemic interactions can collectively reinforce the persistence of the disease as a distributed system.

Rather than being a simple accumulation of local aggregates, the disease unfolds as a distributed system of pathological assemblies, in which deposits act as interconnected nodes, propagating their structural template and potentially reinforcing each other through molecular seeding and cross‐talk (Jucker and Walker 2013; Brettschneider et al. 2015; Brundin et al. 2010; Prusiner 2013; Frost and Diamond 2010). In Alzheimer's disease, for instance, amyloid‐β plaques and tau tangles not only persist locally but also spread along neural circuits, reshaping the functional architecture of the brain (Brettschneider et al. 2015; Hardy and Selkoe 2002). This remodeling is not merely a consequence but may in turn favor further disease progression. In this sense, when multiple aggregates collectively contribute to such network‐level alterations that promote persistence and spread, the process can be interpreted as a form of collective behavior. Similarly, in Parkinson's disease, α‐synuclein aggregates propagate from the gut or olfactory bulb to the brain via the vagus nerve, consistent with the Braak staging model (Brundin et al. 2010; Braak et al. 2003). These findings highlight that protein aggregation disorders represent paradigmatic examples of network pathologies, sustained by structural persistence, systemic dissemination, and emergent collective dynamics.

### 3.3 Fibroses and Systemic Sclerosis

Chronic fibrotic diseases exemplify how pathological organization can transcend individual organs and manifest as systemic, distributed processes. Fibrosis of the liver, lung, kidney, or skin involves the excessive deposition of extracellular matrix (ECM), leading to tissue stiffening, architectural remodeling, and the emergence of self‐perpetuating fibrotic nodules (Wynn 2008; Rockey et al. 2015). These lesions are not inert scars: activated fibroblasts and myofibroblasts maintain a positive feedback loop of ECM production, reinforced by inflammatory mediators and mechanical stress (Henderson et al. 2020). Over time, fibrotic tissue resists resolution, creating stable structures that persist and expand within affected organs.

Systemic sclerosis represents a paradigmatic case where fibrosis operates as a distributed pathological architecture. Here, multiple organs, including skin, lungs, kidneys, and the vascular system, are simultaneously affected (Varga 2008; Denton and Khanna 2017). Global signaling pathways, particularly transforming growth factor‐β (TGF‐β), connective tissue growth factor (CTGF), and interleukin‐6 (IL‐6), act as systemic connectors, ensuring that fibrotic foci emerge, stabilize, and reinforce each other across sites (Gabrielli et al. 2009; Abraham et al. 2025). This represents a particularly clear example of distributed connectivity, with well‐documented systemic mediators. In other pathological systems discussed above and below (e.g., metastases, amyloidoses, autoimmune diseases), the evidence for such global connectors is growing but often less direct, relying on circulating cells, misfolded proteins, or immune mediators that may not achieve the same level of systemic integration as observed in fibrosis. Importantly, mechanical cues arising from ECM stiffening further amplify fibroblast activation, creating a vicious cycle that locks tissues into pathological remodeling states (Hinz 2010). Although systemic or local perturbations, such as immune mediators or inflammatory triggers, may initiate these processes, the distinctive feature of fibrotic diseases lies in their capacity to become self‐sustaining through mechano‐biological feedback loops that persist independently of the initial trigger.

Rather than being reducible to a localized failure of organ maintenance, fibrosis and systemic sclerosis reflect network‐level pathologies, where multiple sites communicate through soluble factors, immune signals, and mechanical stress fields. This perspective highlights their resilience and persistence: once established, fibrotic structures act as interconnected nodes within a broader pathological system, challenging therapeutic attempts aimed only at local damage control.

### 3.4 Chronic Autoimmune Diseases

In autoimmune syndromes such as rheumatoid arthritis (RA), multiple joints become inflamed in parallel, with each inflammatory site functioning as a self‐organizing microenvironment. Within these foci, infiltrating immune cells such as macrophages, dendritic cells, T and B lymphocytes establish local niches that sustain inflammation, promote tissue remodeling, and drive pain responses (Smolen et al. 2016; McInnes and Schett 2011). Each lesion can maintain a degree of autonomy, with synovial fibroblasts and resident immune cells perpetuating chronic activity through local cytokine loops, even in the absence of systemic input (Bartok and Firestein 2010).

Yet, these nodes are not independent: systemic flows of autoantibodies, circulating T cells, and soluble mediators such as TNF‐α, IL‐6, or IL‐17 interconnect the inflammatory sites, ensuring that the disease unfolds as a distributed pathological system (Firestein and McInnes 2017). Importantly, the persistence of disease despite removal of individual joints or localized treatments illustrates that RA is maintained by this systemic immune network rather than by any single focal lesion (Weyand and Goronzy 2021). This distributed nature of pathology is further supported by observations of synovitis emerging in new joints even after effective local interventions, pointing to continuous systemic drivers and immune memory mechanisms (Humby et al. 2019).

This perspective underscores that chronic autoimmune diseases are not merely collections of isolated inflammatory events, but rather network‐like diseases in which multiple self‐sustaining lesions are dynamically linked by circulating immune mediators and autoantibodies. While it is well established that a local perturbation can trigger a systemic inflammatory response that subsequently affects multiple sites, our framework emphasizes that once several inflammatory foci are established, they may collectively reinforce one another and sustain the pathology beyond the initial trigger. In this sense, autoimmune diseases can behave not only as hierarchical systemic responses but also as distributed pathological systems, conceptually aligned with amyloidoses or metastatic cancers, where persistence arises from the reciprocal interactions among multiple pathological sites rather than from a single dominant locus.

### 3.5 Granulomatous Diseases

Granulomas in tuberculosis, sarcoidosis, and other chronic inflammatory conditions represent another form of distributed pathology. Each granuloma constitutes a highly organized microenvironment, where macrophages, epithelioid cells, multinucleated giant cells, and lymphocytes collectively act to wall off persistent pathogens or irritants (Russell et al. 2010; Ramakrishnan 2012). Far from being passive scars, granulomas are dynamic structures that balance host defense and pathogen survival. *Mycobacterium tuberculosis* , for example, can persist within granulomas in a state of latency, while simultaneously exploiting granuloma remodeling to spread and seed new foci (Flynn and Chan 2001; Cadena et al. 2016).

Crucially, granulomas do not occur in isolation. Multiple lesions typically arise across different organs (lung, lymph nodes, skin, liver, spleen), and are interconnected by systemic immune responses, circulating cytokines, and in infectious cases, by dissemination of the pathogen itself (Pagán and Ramakrishnan 2018). In sarcoidosis, for instance, non‐caseating granulomas appear in multiple tissues, including lung, skin, and heart, forming a constellation of inflammatory sites maintained by a shared systemic immune activation (Iannuzzi and Rybicki 2007). Similarly, in tuberculosis, the course of disease depends on the balance between containment and breakdown across populations of granulomas, each with variable microenvironments and evolutionary trajectories (Lenaerts et al. 2015; Gideon and Flynn 2011).

This systemic perspective reframes granulomatous diseases as populations of autonomous but interconnected lesions, rather than as single focal pathologies. Disease persistence, relapse, and treatment resistance often emerge not from one dominant lesion but from the collective dynamics of many granulomas, each capable of self‐sustaining inflammation while contributing to the overall distributed pathology. This view also has important therapeutic implications: targeting systemic mediators (such as TNF‐α or IFN‐γ) or disrupting granuloma organization may be more effective than treating isolated lesions, since the disease network as a whole sustains pathology (Zumla et al. 2013; O'Garra et al. 2013).

These examples highlight a common theme: distributed pathological systems emerge when local pathological units reinforce one another through systemic flows or shared functions. Whether involving cells, tissues, or proteins, these networks exhibit coherence without centralization, persistence without hierarchy, and robustness through connectivity.

## 4 Ecological and Evolutionary Parallels

The dynamics observed in metastatic cancers and other distributed pathologies closely echo organizational patterns that are well recognized in ecology and evolutionary biology. In many natural systems, functional coherence emerges from decentralized interactions, without the need for a central organizing entity. Such analogies help situate distributed diseases within a broader conceptual framework, emphasizing that their persistence relies on principles deeply rooted in evolutionary dynamics.

### 4.1 Superorganisms and Collective Animal Behavior

Social insects such as ants, termites, or honeybees exemplify superorganisms: although no single individual centrally directs the colony, coordinated functions emerge through a combination of decentralized local interactions and hierarchical effectors, such as pheromonal cues or caste‐specific behaviors. These mechanisms allow the colony to achieve highly integrated functions, including foraging, defense, thermoregulation, and reproduction, despite the absence of a central controlling organism (Seeley 1989; Hölldobler and Wilson 2009). Local interactions, mediated by pheromones or simple behavioral rules, generate coherent global outcomes, a phenomenon often described as *emergent collective intelligence* (Camazine et al. 2003). Similarly, metastatic tumors or autoimmune lesions may behave as distributed collectives, where each lesion or inflammatory focus acts autonomously but contributes to the persistence and resilience of the system as a whole (Thomas and Dujon 2025; Korolev et al. 2014). Nevertheless, in contrast to the biological example mentioned above, where the behaviors are genetically programmed and heritable, the outcome of the interactions among entities within these distributed pathologies is not genetically/developmentally programmed and heritable but rather emerges through self‐organization in each individual.

### 4.2 Clonal Networks in Plants

Many clonal plants, including aspen ( *Populus tremuloides* ) or seagrasses ( *Posidonia oceanica* ), persist as networks of interconnected ramets (Harper 1977; Arnaud‐Haond et al. 2007). While each module can survive independently, the clonal network persists through physiological integration, resource sharing, and cooperative buffering against disturbances. Distributed pathologies mirror this logic (with the caveat mentioned above): metastatic nodules, fibrotic foci, or ectopic endometriotic lesions act as “ramets” of a pathological clone, benefiting from redundancy and systemic connectivity that enhance resilience.

### 4.3 Biofilms and Microbial Consortia

Bacterial biofilms provide another powerful analogy. They arise from spatially dispersed cells that coordinate through quorum sensing and extracellular matrix secretion, forming highly structured, cooperative communities (Costerton et al. 1999; Nadell et al. 2016). These consortia exhibit emergent properties such as antibiotic tolerance and resistance to immune clearance, which cannot be reduced to the behavior of single cells. Amyloid deposits, fibrotic nodules, or granulomas resemble such pathological biofilms: autonomous units collectively generate a resilient, self‐sustaining system through mutual reinforcement. In both cases, persistence results from decentralized (self‐organized) cooperation rather than centralized/programmed control.

### 4.4 Ecosystem‐Level Networks

Ecosystems themselves (and the Earth itself) often lack centralized regulation, yet core functions such as nutrient cycling, trophic stability, and resilience to perturbations emerge from distributed interactions among multiple species (see Doolittle 2024). For example, food webs maintain stability not because of a dominant species but through complex feedbacks among producers, consumers, and decomposers (May 2001; Dunne et al. 2002). Similarly, mutualistic networks, such as those linking plants and pollinators or corals and symbiotic algae, persist through decentralized exchanges of resources and signals, creating resilient but fragile architectures (Bascompte and Jordano 2007). Even microbiomes, from the human gut to soil communities, achieve homeostasis via interactions among diverse taxa, where collective functions (e.g., digestion, immune modulation, nutrient cycling) cannot be reduced to any single species (Coyte et al. 2015). In pathology, distributed lesions resemble these ecological systems: they sustain themselves through local feedbacks and systemic flows, such as circulating cytokines, exosomes, or misfolded proteins. Functional stability emerges not from a central driver or program but from the organization of components into configurations that favor persistence of the network as a whole.

## 5 Conceptual Framework and Implications

Taken together, the ecological and evolutionary parallels reviewed above show that distributed, self‐organizing pathologies are not anomalies but manifestations of a general principle: whenever local units organize into configurations that allow interactions in ways that enhance their persistence, selection for function can operate at the collective level. This perspective motivates the need for an explicit conceptual framework capable of capturing how such emergent pathological systems form, stabilize, and resist disruption. In the following section, we outline the criteria that define distributed functional pathologies and derive key implications for evolutionary medicine.

To conceptualize this, we propose that a pathology qualifies as a distributed functional system when three conditions are met:

1. **Autonomous local units:** Each lesion, deposit, or aggregate must be capable of sustaining itself at least temporarily (e.g., a metastatic nodule, an amyloid plaque, a fibrotic focus). These units display internal organization and functional coherence at their level, but they do not reproduce.
2. **Connectivity and systemic flows:** The units are not isolated: they can organize into various configurations and exchange signals, cells, or molecular resources through systemic channels such as circulation, hormonal signaling, or immune responses. Connectivity ensures that local dynamics are coupled, reinforcing the persistence of the whole system.
3. **Emergent collective persistence:** The distributed system exhibits collective properties that go beyond the sum of its parts. The pathology persists not merely because individual lesions are resilient, but because their collective organization stabilizes the disease. In other words, the “fitness” (in terms of persistence) of the pathological system derives from network‐level functions, although some individual lesions or structures may still progress independently of the collective.

These three conditions directly map onto the conceptual equivalencies proposed by Wong et al. (Wong et al. 2023) for evolving systems subject to selection for function. *Autonomous local units* correspond to persistent, interacting components; *connectivity and systemic flows* provide the mechanisms through which multiple configurations of these components can be explored; and *emergent collective persistence* captures selection for function as differential persistence at the level of the distributed system.

To avoid potential misunderstandings, it is important to clarify the nature of selection and of the units on which it operates in distributed pathological systems. Importantly, selection for function does not imply intention, agency, or deliberate strategy on the part of pathological components. In many distributed pathological systems, the relevant units of selection are not individual cells acting “for” a function, but higher‐order configurations, interaction patterns, or organizational states that organize and persist because of their functional consequences at the system level in terms of enhancing its stable and dynamic persistence and novelty generation. In this context, mechanisms such as passive diffusion, templated aggregation, or mechanically constrained spread are not opposed to selection for function; rather, they constitute the generative processes through which alternative configurations arise. Selection for function operates when some of these configurations, whether produced by diffusion, infection, or other processes, are preferentially stabilized because they enhance persistence, propagation, or resistance at the level of the distributed system. This distinction is particularly relevant for neurological and degenerative disorders. For example, the spread of amyloid or other aggregates along neural circuits need not confer a direct benefit to individual neurons, nor reflect a deliberate strategy. Instead, certain patterns of aggregation and connectivity may persist and expand because they stabilize a pathological organization, regardless of their deleterious effects on the host. While infectious etiologies may contribute to such dynamics in some cases, our framework does not presuppose a specific underlying cause but rather focuses on the emergent evolutionary logic governing the persistence of distributed pathological organizations. Note that the selection for function concept is also applicable to (i.e., can include) pathological systems that are composed of reproducing entities as they meet the requirements of being composed of many components that can assume different configurations and can be under selection.

## 6 Implications for Evolutionary Medicine

The proposed framework has profound implications for evolutionary medicine, as it suggests that many pathological processes are best understood not as isolated dysfunctions, but as emergent self‐organizing systems governed by general principles of organization and persistence. A first implication is the identification of a unifying principle: a wide spectrum of conditions, ranging from metastatic cancers to autoimmune syndromes and protein aggregation diseases such as amyloidosis, can be interpreted as manifestations of *distributed system persistence*, whereby pathological units achieve survival and expansion through collective organization. Second, it highlights the value of an explicitly evolutionary perspective, showing that pathologies recruit the same mechanisms of self‐organization, cooperation, and network formation that underpin ecological resilience, microbial communities, and the functional integration of superorganisms. This convergence implies that diseases can be studied with tools derived from ecology and systems biology, such as network theory, tipping‐point analysis, and the study of cooperative cheating dynamics. Third, the framework calls for a new taxonomy of diseases: rather than classifying them exclusively by anatomical site or proximate etiology, we may distinguish between *localized functional pathologies* (e.g., primary tumors, isolated fibromas, single‐site infections) and *distributed functional pathologies* (e.g., metastases, systemic fibroses, disseminated endometriosis), the latter resembling ecological invasions or multicentric breakdowns of regulatory systems. Such a reclassification offers not only conceptual clarity but also therapeutic potential, as it shifts the focus from targeting individual cells or lesions to disrupting the emergent architectures that sustain pathological networks. In this sense, evolutionary medicine is enriched by viewing health and disease through the lens of collective organization and systemic vulnerability, emphasizing that pathological persistence is not random but follows universal evolutionary and ecological rules.

This distinction between localized and distributed functional pathologies can be further interpreted in light of the different modes of selection for function described by (Wong et al. 2023), namely selection for static persistence, dynamic persistence, and novelty generation. While all three modes may operate to some extent in both localized and distributed pathologies, their relative importance is likely to differ. Localized pathologies often rely primarily on static or dynamic persistence, enabling them to withstand host defenses or therapeutic perturbations without substantially reorganizing their functional architecture. By contrast, distributed pathologies combine dynamic persistence with an enhanced capacity for novelty generation, arising from their connectivity, modularity, and systemic reconfigurations. Through the rearrangement of interactions among lesions, deposits, or inflammatory foci, such systems can explore new organizational states that promote persistence, dissemination, or therapeutic resistance.

This interpretation is consistent with clinical observations showing that distributed diseases, such as metastatic cancers, systemic fibroses, or disseminated autoimmune syndromes, are often more difficult to eradicate than localized pathologies, not only because of their spatial extent, but also because of their intrinsic capacity to adapt at the network level. Importantly, we do not suggest that novelty generation is absent from localized pathologies, but rather that distributed organization amplifies its role by increasing the space of possible configurations on which selection for function can act.

## 7 Implications for Therapy

Clinically, this perspective emphasizes that targeting individual lesions or focal sites of pathology may often be insufficient if the disease is sustained by processes operating at the level of distributed networks. Although chemotherapies and modern immunotherapies already act systemically and are not restricted to local interventions, many treatment strategies still focus primarily on controlling individual lesions or shrinking dominant sites of disease. A distributed‐system view suggests that truly effective interventions may also need to disrupt the connectivity, communication channels, and feedback loops that allow pathological networks to maintain coherence across multiple sites. In cancer, for example, surgical resection or local ablation can remove primary tumors, but relapse and metastasis frequently occur because circulating tumor cells (CTCs) and their microenvironmental interactions continue to sustain systemic progression (e.g., Aceto et al. 2014; Massagué and Obenauf 2016; Li et al. 2017; Fu et al. 2022). Similarly, in autoimmune syndromes or neurodegenerative diseases, local treatments may fail if the pathology persists through network‐level propagation, such as cytokine storms or prion‐like spreading of misfolded proteins (Prusiner 2013; Soto and Pritzkow 2018). From this standpoint, new therapeutic strategies can be envisioned. Importantly, these system‐level approaches should be viewed as complementary to existing therapies rather than as replacements, and their potential trade‐offs, such as increased susceptibility to infection or malignancy when targeting immune mediators, must be carefully considered, as already well documented in clinical practice. One approach is to **disrupt connectivity**, for instance by blocking the dissemination of CTC clusters, neutralizing systemic cytokine signals, or preventing the templated spread of pathological proteins. Another approach is to **destabilize network coherence**, achieved by interfering with the feedback loops, trophic dependencies, or shared metabolic resources that allow distributed lesions to maintain collective resilience (Korolev et al. 2014; Thomas, Capp, et al. 2025). Finally, this framework calls for the development of **system‐level biomarkers**, capable of monitoring not only focal lesions but also the degree of pathological connectivity across tissues and organs, for example, through circulating DNA/RNA signatures, extracellular vesicles, or imaging of systemic fibrosis (Cristiano et al. 2019; Mathios et al. 2021; Foda et al. 2023; Pantel and Alix‐Panabières 2019). In sum, the concept of *selection for function* in distributed systems reframes distributed pathologies as a challenge of network‐level organization. By identifying the ecological and evolutionary conditions that allow such systems to emerge and persist, we open the way to therapeutic strategies that are more integrative, systemic, and ultimately capable of destabilizing disease persistence at its roots.

Recent theoretical work on selection for function has emphasized the role of novelty generation and state‐space expansion in the emergence of new functional organizations, notably through the concept of selective funneling (Wong et al. 2025). In this framework, evolving systems transition between distinct functional landscapes as new components arise, new interactions become possible, or new selection pressures come into play. Tumor progression has already been interpreted through this lens (Thomas et al. 2024), highlighting how early functional innovations can funnel the system toward increasingly resilient pathological states.

This perspective may also apply to other distributed functional pathologies discussed here. Before reaching a fully stabilized network architecture, pathological systems may pass through early, plastic phases characterized by high exploratory potential, during which novelty generation enables the discovery of configurations that enhance persistence, dissemination, or resistance. From a therapeutic standpoint, this suggests a complementary strategy: beyond disrupting established pathological networks, interventions could aim to constrain or delay novelty generation itself, thereby preventing distributed pathologies from accessing functionally advantageous organizational states. Such approaches might be particularly relevant at early stages of disease development, when pathological systems remain malleable and have not yet been funneled into deeply stable configurations.

## 8 Conclusion

The framework of selection for function provides a powerful lens through which to reinterpret the organization and persistence of many pathological processes. While initially developed to account for the collective behavior of tumor cells and the progression of metastasis, this perspective also applies to a much broader class of diseases. Conditions such as endometriosis, amyloidoses, fibroses, autoimmune syndromes, and granulomatous infections can all be understood as distributed pathological systems, where multiple local units interact to sustain a higher‐level functional organization.

Crucially, these systems illustrate that pathological cohesion does not require a single, dedicated central controller or program. Although systemic signals such as cytokines or hormones can act as hierarchical effectors that influence multiple sites simultaneously, they do not function as central organizers in the sense of a coordinating entity directing the behavior of all pathological units. Rather, as in superorganisms, biofilms, or clonal plant networks, coherence emerges from the interactions among distributed components, allowing pathologies to persist and thrive through network‐level connectivity and emergent functions. This recognition unifies seemingly disparate conditions under a common evolutionary principle: whenever local pathological units can organize, interact and reinforce one another, selection for function can act at the network level, ensuring persistence despite host defenses or therapeutic interventions.

This shift in perspective has profound implications. Conceptually, it reframes disease not simply as a local breakdown at local sites but as a network‐level phenomenon, aligning pathology with general principles of ecology and evolution. Clinically, it calls for therapies that do not only attack individual lesions but also disrupt the flows, signaling, and feedback loops that give distributed pathologies their coherence and stability.

Moreover, many distributed pathologies may emerge from processes that evolved as adaptations for the host. Wound healing, fibrosis, granuloma formation, immune diversification, or protein conformational changes are all beneficial strategies to contain damage, repair tissue, or enhance defense. Yet if expressed in atypical contexts, these processes may acquire a degree of autonomy from the central controller (i.e., the organism level) through selection for function, evolving into persistent, network‐level organizations that ultimately compromise host health. This duality underscores the evolutionary ambiguity of such systems: what begins as a fitness‐enhancing response under organismal‐level control can, under certain conditions, become a self‐organized and self‐sustaining pathology under selection for function at its level. Recognizing this transition from adaptive processes to maladaptive traits adds an important dimension to the study of distributed diseases.

Looking forward, we argue that understanding selection for function acting within individual organisms to maintain pathological systems should become a research priority in evolutionary medicine. By systematically identifying and characterizing such pathologies, and by designing strategies to destabilize their systemic coherence, we may uncover novel ways to control conditions that have long resisted focal interventions. In this sense, distributed pathologies are not exceptions, but rather exemplify a fundamental mode of pathological organization at the system level, one that demands new ways of thinking about health, disease, and therapy.

## Funding

This work was funded by the CNRS (IRP CANECEV), the HOFFMANN Family, and by the following grant: EVOSEXCAN project (ANR‐23‐CE13‐0007).

## Conflicts of Interest

The authors declare no conflicts of interest.

## Data Availability

The authors have nothing to report.
